# mTORC1 signaling pathway integrates estrogen and growth factor to coordinate vaginal epithelial cells proliferation and differentiation

**DOI:** 10.1038/s41419-022-05293-8

**Published:** 2022-10-11

**Authors:** Shuo Wan, Yadong Sun, Jiamin Fu, Hongrui Song, Zhiqiang Xiao, Quanli Yang, Sanfeng Wang, Gongwang Yu, Peiran Feng, Wenkai Lv, Liang Luo, Zerong Guan, Feng Liu, Qinghua Zhou, Zhinan Yin, Meixiang Yang

**Affiliations:** 1 The First Affiliated Hospital, Jinan University, Guangzhou, Guangdong 510632 China; 2 Guangdong Provincial Key Laboratory of Tumor Interventional Diagnosis and Treatment, Zhuhai Institute of Translational Medicine Zhuhai People’s Hospital Affiliated with Jinan University, Jinan University, Zhuhai, 519000 Guangdong China; 3 The Biomedical Translational Research Institute, Faculty of Medical Science, Jinan University, Guangzhou, 510632 Guangdong China; 4 Guangdong Women and Children Hospital, Guangzhou, Guangdong 510010 China; 5 Department of Medical Genetics, Zhongshan School of Medicine, Sun Yat-sen University, Guangzhou, 510080 China

**Keywords:** Experimental models of disease, Reproductive disorders

## Abstract

The mouse vaginal epithelium cyclically exhibits cell proliferation and differentiation in response to estrogen. Estrogen acts as an activator of mTOR signaling but its role in vaginal epithelial homeostasis is unknown. We analyzed reproductive tract-specific *Rptor* or *Rictor* conditional knockout mice to reveal the role of mTOR signaling in estrogen-dependent vaginal epithelial cell proliferation and differentiation. Loss of *Rptor* but not *Rictor* in the vagina resulted in an aberrant proliferation of epithelial cells and failure of keratinized differentiation. As gene expression analysis indicated, several estrogen-mediated genes, including *Pgr* and *Ereg* (EGF-like growth factor) were not induced by estrogen in *Rptor* cKO mouse vagina. Moreover, supplementation of EREG could activate the proliferation and survival of vaginal epithelial cells through YAP1 in the absence of *Rptor*. Thus, mTORC1 signaling integrates estrogen and growth factor signaling to mediate vaginal epithelial cell proliferation and differentiation, providing new insights into vaginal atrophy treatment for post-menopausal women.

## Introduction

Vaginal epitheliums serve as a physical barrier that protects the vaginal cavity from external harm and pathogen invasion. Vaginal epitheliums exhibit cyclical, estrogen-dependent cell proliferation and differentiation during the estrous cycle, and estrogen administration induces vaginal epithelial cell proliferation in ovariectomized (OVX) mice. However, the mechanisms governing cell proliferation and differentiation in the vagina remain poorly understood. At the onset of menopause, estrogen level rapidly decreases, which has pronounced effects on the vagina, accelerating its natural process of atrophy [1]. Estrogen replacement therapy is often beneficial in treating vaginal atrophy. However, estrogen replacement therapy has undesired side effects, the most serious of which is an increased risk of cancer [2]. Therefore, vaginal homeostasis should be separately regulated, and effective and safe agents which positively affect the underlying physiology and thus improve the qualitative aspects of vaginal homeostasis in post-menopausal women would be valuable.

In rodent reproductive organs such as the vagina and uterus, estrogen plays a regulatory role by binding to stromal expressed estrogen receptor α (ERα) [3–5]. Estrogen-induced stromal-secreted growth factors activate cell signal transduction through PI3K/Akt and mitogen-activated protein kinase (MAPK) signal cascade to promote epithelium proliferation [6–10]. Aberrant PI3K/Akt activation results in complex atypical hyperplasia and endometrioid carcinoma in the uterus [11–15]. These findings suggested that PI3K/Akt signaling pathways are functional mediators of estrogen-induced cell proliferation and differentiation. The mammalian target of rapamycin (mTOR) is an important component of the PI3K/AKT pathway. mTOR is a serine/threonine protein kinase that associates with the scaffold proteins Raptor and Rictor respectively in two distinct complexes, mTOR complex 1 (mTORC1) [16], which promotes protein synthesis and RNA transcription primarily by phosphorylating S6, and mTOR complex 2 (mTORC2) [17] that activates S473 site of Akt [18].

mTOR plays a central role in sensing environmental conditions and regulating almost all aspects of metabolism at both the cellular and organismal levels [19]. Dysregulation of mTOR has been observed in various human pathological conditions, such as cancer [20], neurological diseases [21], diabetes [22], cardiovascular complications [23], and aging [21]. Further, targeting mTOR is one of the most promising fields for the efficient treatment of these diseases. Recently, accumulating lines of evidence have discovered the role of mTOR in female reproduction under physiological and pathological conditions, including embryonic development [24, 25], ovarian somatic cell proliferation [26], folliculogenesis [27], oocyte meiotic maturation [28], ovarian aging [29], fertility [29], puberty onset [30], and uterus endometrium homeostasis [31]. However, little is known about the role of mTOR signaling or how it interacts with estrogen signaling in the vagina. The role of mTOR signaling pathway in vaginal epithelial homeostasis, especially in the process of vaginal atrophy in menopausal women, needs further investigation.

In this study, we assessed the role of mTOR signaling in estrogen-mediated vaginal epithelial proliferation and differentiation. Using a mice model with a reproductive tract-specific *Rptor* or *Rictor* deletion, we found that loss of *Rptor* but not *Rictor* in the vagina resulted in aberrant proliferation of epitheliums and failure of keratinized differentiation. Remarkably, gene expression analysis showed estrogen unresponsiveness in the vagina of estrogen-primed OVX *Rptor* cKO mice. Several estrogen-mediated genes, including *Pgr* and EGF-like growth factor *Ereg* were not induced by estrogen in *Rptor* cKO mouse vagina. Moreover, supplementation of EREG could activate proliferation and survival of vaginal epitheliums through YAP1 in the absence of Raptor. Our data unveiled the crucial role of mTORC1 signaling in integrating estrogen and growth factor signaling for vaginal epitheliums proliferation and differentiation, providing new insights into how mTOR signaling pathway functions in vagina homeostasis.

## Results

### Estrogen promotes activation of mTORC1 signaling in the vaginal epitheliums

**Fig. 1:**
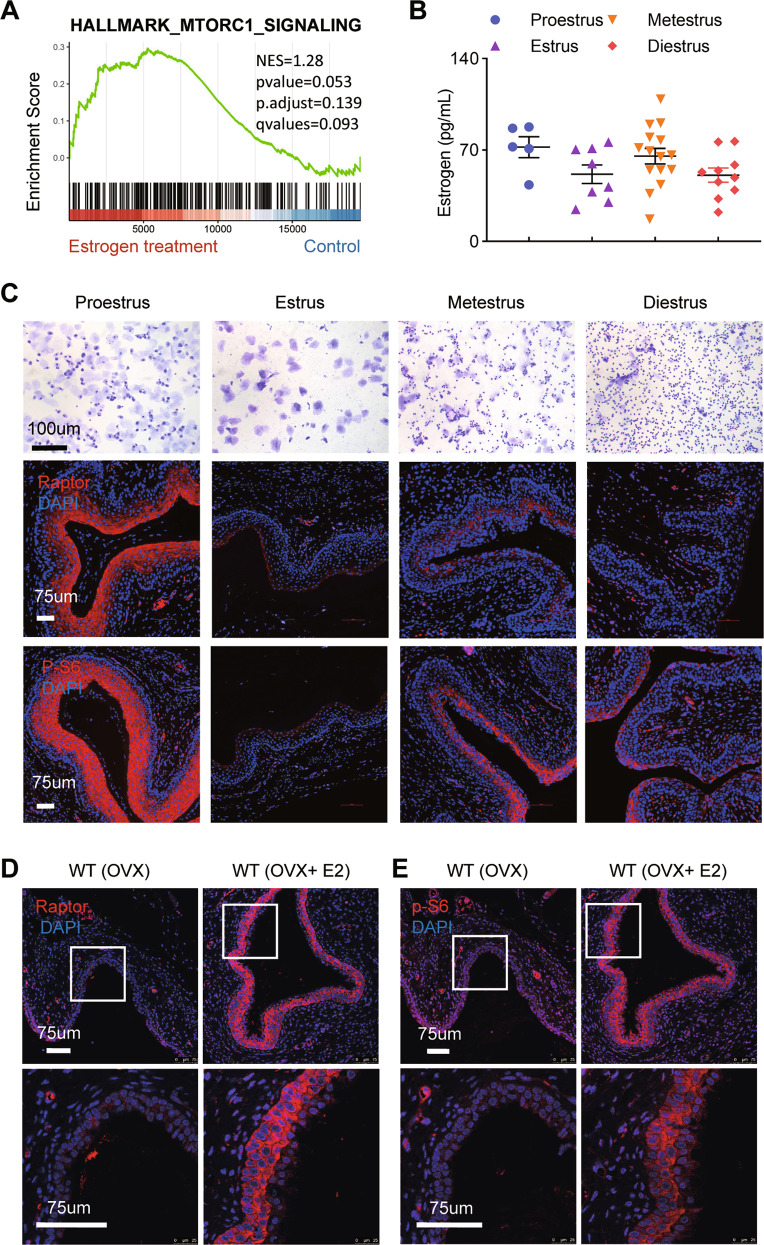
Estrogen mediates activation of mTORC1 signaling in the vaginal epithelium. **A** GSEA enrichment plots of mTORC1 signaling in vaginal biopsies transcriptome from 19 menopausal women with vaginal dryness pre and post 3-month E2 treatment (GSE11622). **B** Serum E2 level in mice during proestrus (*n* = 5), estrus (*n* = 8), metestrus (*n* = 15), and diestrus (*n* = 10). Values are expressed as the mean ± SEM. **C** Cytological assessment of vaginal smears using the crystal violet staining method for estrous cycle determination in 8-wk-old WT mice. *n* = 5 in each group. Microscopy with magnification ×20. Scale bars: 100 μm (Upper). Representative images of the immunofluorescence staining for Raptor (Middle) and Phospho-S6 (Ser235/236) (lower) in mouse vagina during proestrus, estrus, metestrus, and diestrus. *n* = 5 in each group. Nuclei were stained with DAPI. Microscopy with magnification ×20. Scale bars: 75 μm. **D**, **E** Representative images of the Raptor (**D**) or p-S6(Ser235/236) (**E**) immunofluorescence staining in the vagina of OVX mice with sesame oil (*n* = 3) and E2 administration (*n* = 3). The experiments were repeated three times. Nuclei were stained with DAPI. Microscopy with magnification ×20 (Upper) and ×63 (Lower). Scale bars: 75 μm.

According to previous research [32], mTORC1 signaling was enriched in the vagina of menopausal women with 3-month estradiol (E2) treatment by gene set enrichment analysis (GSEA) analysis (Fig. 1A), suggesting a possible role of estrogen in the development and functional maintenance of vagina. To determine the correlation between mTOR activity and estrogen level, we examined the expression of Raptor and p-S6 throughout the menstrual cycle with periodic changes in estrogen levels (Fig. 1B). The results revealed a dynamic activation of mTORC1 signaling during the estrus cycle in mice aged 8–10 weeks. Raptor and p-S6 were highest expressed in vaginal epitheliums during proestrus, when estrogen levels were the highest (Fig. 1C). To avoid any interference of the hypothalamus–pituitary–gonadal axis, we further used OVX mice and analyze the activation of mTORC1 signaling followed by estrogen-injection. The vaginal epithelium and stroma of OVX mice showed extremely low levels of mTORC1 signaling activity. However, when OVX mice were treated with estrogen, mTORC1 signaling was rapidly activated in the vaginal epitheliums, and Raptor protein expression and ribosomal protein S6 phosphorylation were significantly increased (Fig. 1D, E). These results suggested that estrogen mediates the activation of mTORC1 signaling in vaginal epitheliums.

### *Rptor* deficiency led to a disruption of estrous cycle homeostasis

**Fig. 2:**
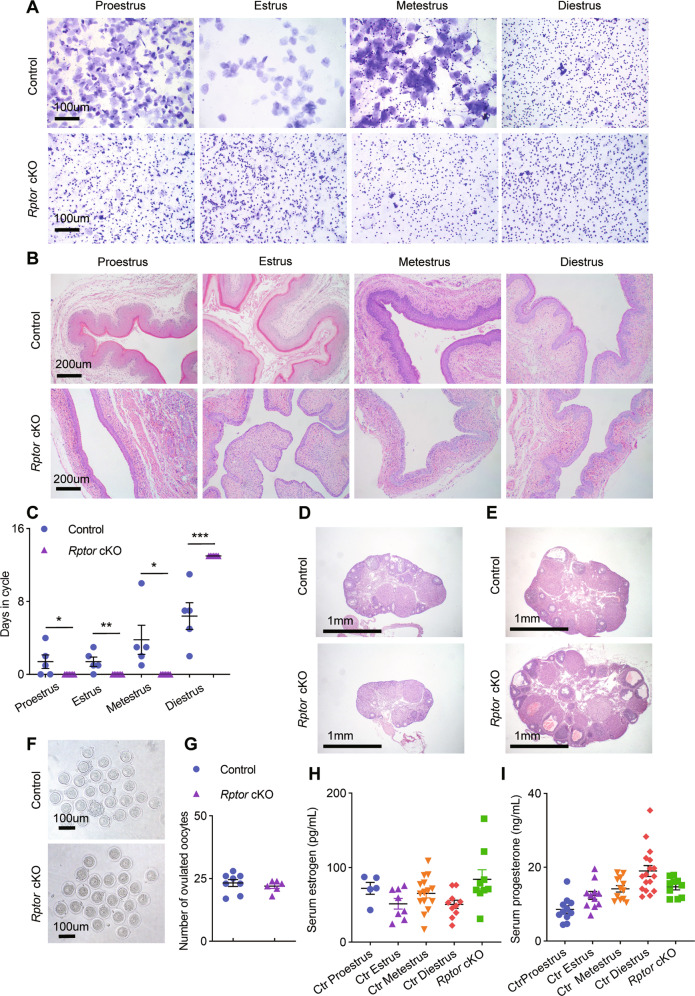
*Rptor* deficiency disrupts estrous cycle homeostasis while maintaining normal ovarian function. **A** Representative images for cytological assessment of vaginal smears of control and *Rptor* cKO mice using crystal violet staining method for estrous cycle determination. *n* = 5 mice in each group. Microscopy with magnification ×20. Scale bars: 100 μm. **B** Morphologic changes in the vaginal mucosa during the mouse estrus cycle by HE staining. *n* = 5 mice at each stage. Microscopy with magnification ×10. Scale bars: 200 μm. **C** Estrous cyclings in 8-week-old control (*n* = 5) and *Rptor* cKO (*n* = 7) mice were determined daily by vaginal lavage cytology for 13 days. Values are expressed as the mean ± SEM. **D** Representative HE-stained ovarian sections of control and *Rptor* cKO mice at 8~10 weeks of age. *n* = 5 mice in each group. Microscopy with magnification ×4. Scale bars: 1 mm. **E** Representative HE-stained ovarian sections of control (*n* = 8) and *Rptor* cKO (*n* = 6) mice at 8~10 weeks of age after superovulation. Microscopy with magnification ×4. Scale bars: 1 mm. **F** Oocytes were collected from the oviducts of superovulated control (*n* = 8) and *Rptor* cKO (*n* = 6) mice. Microscopy with magnification ×10. Scale bars: 100 μm. **G** Scatter plot shows the statistics of oocyte number between the two groups of mice in **F**. Values are expressed as the mean ± SEM. **H**, **I** Serum E2 (**H**) and P4 (**I**) levels in control and *Rptor* cKO mice during proestrus, estrus, metestrus, and diestrus. *n* = 5 (control mice at proestrus stage), *n* = 8 (control mice at estrus stage), *n* = 15 (control mice at metestrus stage), *n* = 10 (control mice at diestrus stage), and *n* = 9 (*Rptor* cKO mice). Values are expressed as the mean ± SEM.

To study the role of mTORC1 in the vagina, we generated a conditional *Rptor* knockout mouse model. To do so, *Rptor* flox/flox mice [33, 34] were mated to mice carrying a *Pgr*-Cre allele, in which Cre protein expression is driven by *Pgr* promoter [35] (Supplemental Fig. 1A). We verified *Rptor* knockout efficiencies at mRNA and protein levels in the vagina by quantitative RT-PCR (qPCR), western blot, and immunofluorescence (Supplemental Fig. 1B-D). Immunofluorescence of p-S6 (Ser235/236) further confirmed the inactivation of mTORC1 signaling in *Rptor* cKO vaginal epitheliums using OVX model (Supplemental Fig. 1E). Since mTORC1 activity changes with the estrus cycle, we examined the effect of *Rptor* deletion on the estrus cycle, determined by daily-vaginal smears for 13 consecutive days and summarized in Fig. 2C. We found the vaginal smears and histology of the vagina were dynamically changing during the estrus cycle in control mice, while *Rptor* cKO mice remained in diestrus, as the vaginal smears of *Rptor* cKO mice contain a lot of leukocytes (Fig. 2A), with an atrophied epithelium of 2–3 cell layers (Fig. 2B).

Given that *Pgr*-Cre is expressed in the granulosa cells of preovulatory follicles [35] and mTORC1 has been implicated in ovarian function [36], we also evaluated the effect of *Rptor* on ovarian function. Histological results of ovarian sections of control and *Rptor* cKO mice at 8~10 weeks of age before (Fig. 2D) and after (Fig. 2E) superovulation showed no differences, with comparable matured preovulatory follicles and corpus luteum. Lastly, *Rptor* cKO and control mice produced equal amounts of E2 and P4 (Fig. 2H, I). So the loss of estrus cycle in *Rptor* cKO mice was not caused by compromised ovarian function, suggesting the important role of mTORC1 signaling in estrous cycle establishment and maintenance.

### *Rptor* deficiency resulted in menopausal-like dysfunction of vagina via promoting cell death and inhibiting cell survival

**Fig. 3:**
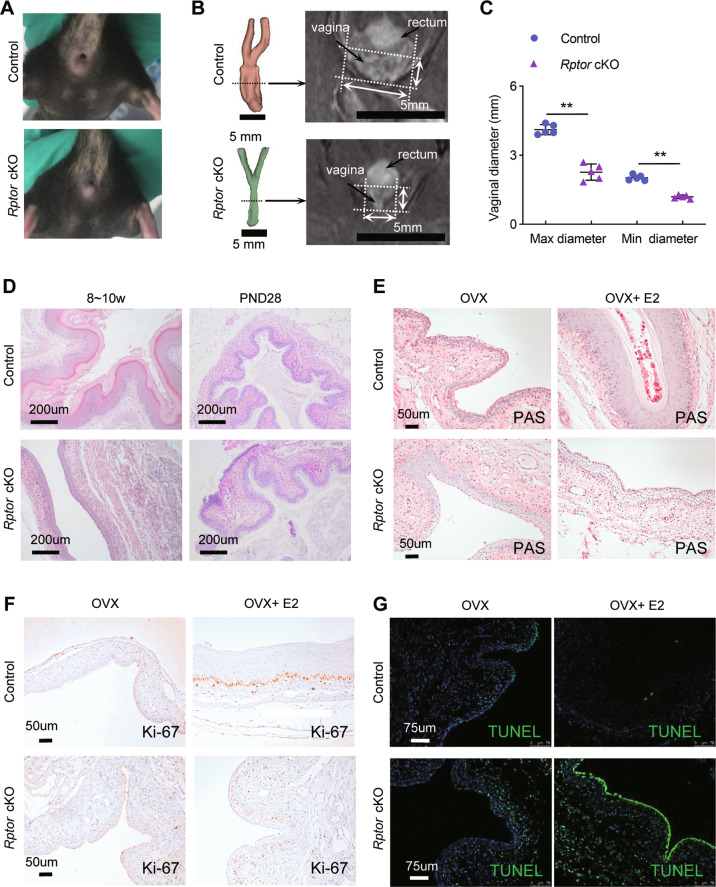
*Rptor* depletion leads to vaginal atrophy via promoting cell death and inhibiting cell proliferation. **A** Representative images of the external vaginal orifice in control (*n* = 5) and *Rptor* cKO (*n* = 5) female mice. **B** 3D renderings of the female reproductive tract in control (*n* = 5) and *Rptor* cKO (*n* = 5) mice based on MRI T2 weighted images. Scale bars: 5 mm. **C** Accurate max and min diameter statistics of the vagina were measured based on **B**. Values are expressed as the mean ± SEM. (**D**) Representative HE-stained vaginal sections of control and *Rptor* cKO mice at 8 weeks (left) (*n* = 5 mice in each group) and postnatal day 28 (right) (*n* = 3 mice in each group). The experiments were repeated 3 times. Microscopy with magnification ×10. Scale bars: 200 μm. **E**, **F** Control and *Rptor* cKO mice were ovariectomized and rested for 2 weeks. E2 was administrated for 3 consecutive days. Representative images of the PAS (**E**) or Ki67 (**F**) staining of the vagina in control and *Rptor* cKO mice. Nuclei were stained with hematoxylin. *n* = 3 in each group. The experiments were repeated 3 times. Microscopy with magnification ×20. Scale bars: 50 μm. **G** Representative images of the TUNEL immunofluorescence staining in the vagina of OVX mice following E2 administration. Nuclei were stained with DAPI. *n* = 3 in each group. The experiments were repeated 3 times. Microscopy with magnification ×20. Scale bars: 75 μm.

The observation of the reproductive duct showed that the external vaginal orifice of *Rptor* deficient mice was significantly smaller than that of control mice (Fig. 3A), and the maximum (max) and minimum (min) vaginal diameter estimation based MRI also decreased in adult *Rptor* cKO mice compared with the control mice (Fig. 3B, C). Intriguingly, loss of keratinization of the vaginal epithelium was observed in adult *Rptor* cKO mice, while no significant difference was found between *Rptor* cKO and control mice on postnatal day 28 (Fig. 3D). Postnatal day 28 represent the begin of prepubertal period, when mouse ovaries are immature, and the hormone levels are low. These results suggested that loss of *Rptor* might lead to menopausal dysfunction of mouse vagina, such as atrophy and loss of estrous cycle, and also indicated that mTORC1 signaling pathway mediates hormonal regulation of vaginal development in adult mice.

In addition, PAS staining revealed a thickened cornified vaginal epithelium of the control mice, but a loss of stratified squamous epithelial cells and mucin production in the vagina of *Rptor* cKO mice treated with E2 (Fig. 3E). We next examined the proliferation of vaginal epitheliums using immunohistochemistry by Ki67. Proliferation levels were lower in the vaginal basal layer cells in *Rptor* cKO mice than in controls treated with E2 (Fig. 3F). We also investigated the apoptosis in vaginal epitheliums using TUNEL. The apoptotic indices were augmented in both vaginal epithelium and stroma in E2-treated *Rptor* cKO mice compared with controls (Fig. 3G), suggesting mTORC1 signaling contributes to regulating the survival of both epithelial and stromal cells. These results suggested a defective response to E2 in the vagina of *Rptor* cKO mice, and *Rptor* depletion leads to vaginal atrophy via promoting cell death and inhibiting cell proliferation.

### *Rptor* depletion impaired the expression of genes involved in the development and differentiation of vaginal epithelium

**Fig. 4:**
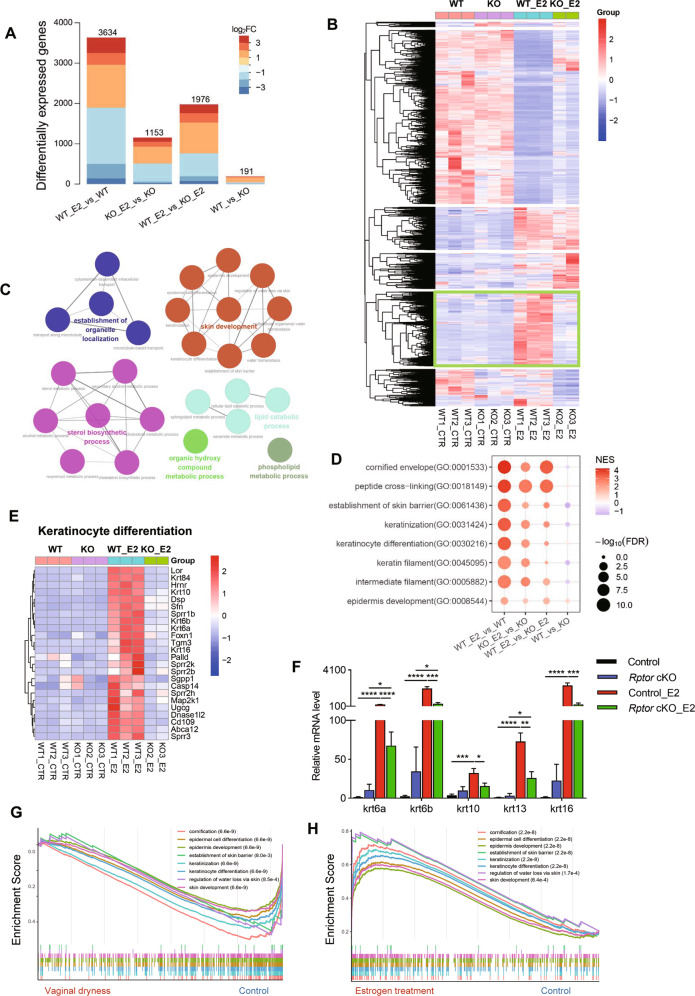
*Rptor* depletion impairs the expression of genes involved in the development and differentiation of vaginal epithelium. **A** RNAseq analysis was performed using the vagina of OVX WT mice and *Rptor* cKO mice with or without 3 day-E2 administration. The number of the differentially expressed genes of the above four groups are plotted. **B** Heatmap of significantly changing genes in the vagina among the above four groups in A belonging to six major classes as indicated. **C** The GO terms of significantly changed genes in the green frame in B were enriched using the Cytoscape plug-in ClueGO. **D** GSEA analysis using the GO terms. **E** Heatmap of the expression level of keratinocyte differentiation-related genes from the significantly changing genes in the green frame in **B**. **F** Expression of *Krt6a*, *Krt6b*, *Krt10*, *Krt13*, *Krt16* was quantified using qPCR in the vagina of the mice indicated in **A**. *n* = 7 (OVX control mice), *n* = 5 (OVX *Rptor* cKO mice), *n* = 6 (OVX control mice treated with E2), *n* = 5 (OVX *Rptor* cKO mice treated with E2). Values are expressed as the mean ± SEM. **G** GSEA enrichment analysis of the transcriptome of vaginal biopsies from post-menopausal women with vaginal dryness (*n* = 4), and post-menopausal controls without vaginal dryness (*n* = 6) (GSE26761). **H** GSEA enrichment analysis of the transcriptome of vaginal biopsies from post-menopausal women suffering from vaginal dryness pre and post 3-month E2 therapy (*n* = 19) (GSE11622).

To avoid confounding factors triggered by hormonal changes throughout the estrus cycle, RNAseq of vaginal tissues was performed using OVX mice in the presence and absence of three daily-E2 injections. 3634 genes were significantly expressed in vagina of control mice after E2 treatment, while 1153 genes were altered in *Rptor* cKO mice after E2 treatment. 1976 genes were differentially expressed in the vagina between *Rptor* cKO and control mice treated with E2, and only 191 genes were differentially expressed in the vagina between *Rptor* cKO and control untreated mice (Fig. 4A). These differentially expressed genes in the above four groups were then divided into six clusters (Fig. 4B). We found that 910 genes in one cluster are specifically elevated in control mice treated with E2, but not in the other three groups (Fig. 4B), that are involved in the establishment of organelle localization, skin development, sterol biosynthetic process, lipid catabolic process, and several metabolic processes (Fig. 4C). Furthermore, GSEA analysis results showed that the top positively enriched pathways in “WT_E2” group compared to “WT” group and “KO_E2” group were related to skin development (Fig. 4D), including cornified envelope, peptide cross-linking, establishment of skin barrier, keratinization, keratinocyte differentiation, etc. Several keratinocyte differentiation-related genes were significantly upregulated in the vagina of control mice treated with E2, but remained unchanged in *Rptor* cKO mice treated with E2 (Fig. 4E). And mRNA levels of keratin family genes, specifically *Krt6a*, *Krt6b*, *Krt10*, *Krt13*, and *Krt16*, were validated by qPCR (Fig. 4F). We also performed GSEA analysis based on previously released datasets (GSE26761 and GSE11622). Notably, cornification-related biological processes were significantly downregulated in the vagina of post-menopausal women suffering from vaginal dryness (Fig. 4G). Three-month E2 treatment also significantly promoted cornification possess promoting vaginal health in menopausal women (Fig. 4H). These results suggested that loss of mTORC1 signaling pathway impaired E2-mediated keratinization-related genes expression and vaginal epithelial differentiation.

### Loss of Raptor compromises estrogen responsiveness and thus PR expression in the vagina

**Fig. 5:**
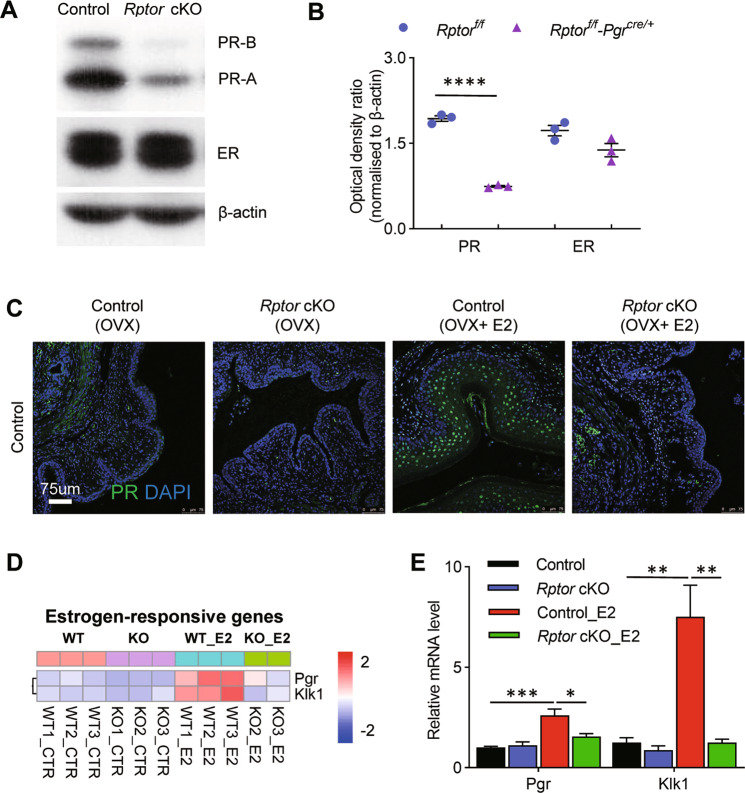
*Rptor* depletion leads to estrogen unresponsiveness of the vagina. **A**, **B** Immunoblotting analysis of PR and ER protein levels in the vagina of control (*n* = 3) and *Rptor* cKO (*n* = 3) mice. β-Actin was used as the loading control. **C** Representative images of the immunofluorescence staining of PR in the vagina of OVX control and *Rptor* cKO mice administrated with E2 for 3 consecutive days. Nuclei were stained with DAPI. *n* = 3 in each group. The experiments were repeated three times. Microscopy with magnification ×20. Scale bars: 75 μm. **D** Heatmap of the expression level of estrogen-regulated target genes in the vagina of control and *Rptor* cKO mice in the presence or absence of E2 administration for 3 days. **E** Estrogen-regulated target genes in **D** were validated by qPCR. *n* = 7 (OVX control mice), *n* = 5 (OVX *Rptor* cKO mice), *n* = 6 (OVX control mice treated with E2), *n* = 5 (OVX *Rptor* cKO mice treated with E2). Values are expressed as the mean ± SEM.

It is reported that mouse vaginal epithelium exhibits cell proliferation and differentiation cyclically in response to E2, through ER [37]. PR, consist of PR-A, and PR-B isoforms, is a classical ER-regulated protein. To look into the underlying mechanism of steroid resistance in *Rptor* cKO mice, we analyzed ER and PR expression in the vagina of *Rptor* cKO and control mice, and found that vaginal PR (PR-A, and PR-B) expression in *Rptor* cKO mice was markedly decreased compared with control mice, while ER level was comparable in *Rptor* cKO and control mice (Fig. 5A, B). We also detected PR expression using OVX mice, and found that PR level was significantly increased after exogenous E2 treatment in control mice, but not in *Rptor* cKO mice (Fig. 5C). To identify whether the decreased PR expression is due to hampered estrogen activity, we also profile ER target genes in our RNAseq data, and *Pgr* and *Klk1* mRNA levels were validated by qPCR (Fig. 5D, E). These results confirmed that *Rptor* deficiency compromised estrogen responsiveness and thus the expression of its target genes including *Pgr*, regardless of the presence of normal circulating E2 level and ER level.

### EREG acts as a potential factor for vaginal epithelial cell proliferation and differentiation downstream of mTORC1 signaling

**Fig. 6:**
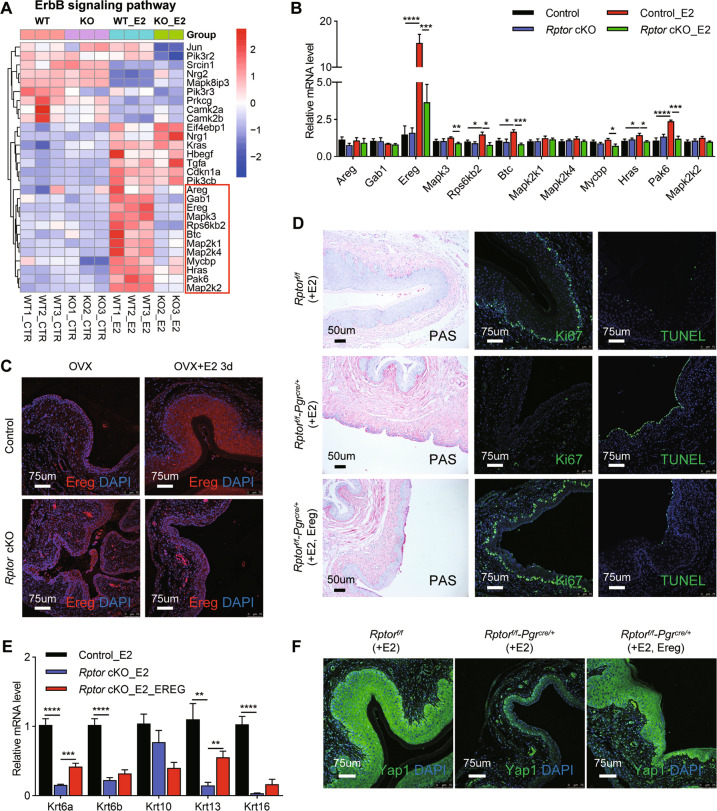
EREG acts as a potential factor for vaginal epithelial cell proliferation and differentiation. **A** Heatmap of the expression levels of ErbB signaling pathway signature genes in the vagina of control and *Rptor* cKO mice in the presence or absence of E2 administration. **B** qPCR was performed to verify the genes in the frame in A. *n* = 7 (OVX control mice), *n* = 5 (OVX *Rptor* cKO mice), *n* = 6 (OVX control mice treated with E2), *n* = 5 (OVX *Rptor* cKO mice treated with E2). Values are expressed as the mean ± SEM. **C** Representative images of the immunofluorescence staining of EREG in the vagina in OVX control and *Rptor* cKO mice administrated with or without E2. Nuclei were stained with DAPI. Microscopy with magnification ×20. Scale bars: 75 μm. **D** OVX control and *Rptor* cKO mice were administrated with E2 and/or EREG. The vaginas were harvested for PAS staining, Ki67 immunofluorescence staining, and TUNEL staining. In PAS staining, nuclei were stained with hematoxylin. Scale bars: 50 μm. In Ki67 immunofluorescence staining and TUNEL staining, nuclei were stained with DAPI. Microscopy with magnification ×20. Scale bars: 75 μm. **E** Expression levels of *Krt6a*, *Krt6b*, *Krt10*, *Krt13*, *Krt16* in the vagina of the mice were quantified using qPCR. *n* = 6 (OVX control mice treated with E2), *n* = 5 (OVX *Rptor* cKO mice treated with E2), *n* = 4 (OVX *Rptor* cKO mice treated with E2 and EREG). Values are expressed as the mean ± SEM. **F** The vaginas as indicated in **D** were harvested for immunofluorescence staining of YAP1, nuclei were stained with DAPI. *n* = 6 (OVX control mice treated with E2), *n* = 5 (OVX *Rptor* cKO mice treated with E2), *n* = 4 (OVX *Rptor* cKO mice treated with E2 and EREG). The experiments were repeated three times. Microscopy with magnification ×20. Scale bars: 75 μm.

It has been reported that the coordinated crosstalk between the ErbB signaling pathway and ERα contributes to the estrogen effect in the female reproductive organ, mediated by several EGF-like growth factors including EGF, TGF-α, HB-EGF, BTC, AREG, EREG, and NRGs. The hallmark genes of ErbB signaling pathway were superimposed onto our RNAseq datasets and profiled in Fig. 6A. 12 genes were specifically elevated in OVX control mice treated with E2, but not in the other three groups, indicating their possible important functions. The mRNA levels of the 12 genes were then validated by qPCR, with *Ereg* being the most significant one (Fig. 6B). Remarkably, EREG was exclusively expressed in the vaginal epithelium in E2-primed OVX control mice, but not in the *Rptor* cKO mice as the immunofluorescence results showed in Fig. 6C, implying a possible autocrine mechanism for E2-mTORC1-EREG signaling in the vaginal epithelium.

To elucidate the role of EREG in the vaginal keratinocyte differentiation in *Rptor* cKO mice, control and *Rptor* OVX mice were administrated with E2 and/or EREG. PAS-positive cells were observed in the superficial cells in the vagina of control mice treated with E2. In contrast, only a few PAS-positive cells were observed in the vagina from *Rptor* cKO mice treated with E2 and/or EREG. Intriguingly, co-administration of EREG and E2 resulted in a thickening of the vaginal epithelium in OVX *Rptor* cKO mice as in OVX control mice treated with E2 (Fig. 6D). Moreover, EREG and E2 co-administration enhance vaginal epithelial cell proliferation and maintain cell survival of *Rptor* cKO mice as E2-treated control mice revealed by Ki67 and TUNEL staining (Fig. 6D). Subsequently, keratinocyte differentiation-related genes including *Krt6a*, and *Krt13* levels were upregulated in the vagina of OVX *Rptor* cKO mice after EREG administration (Fig. 6E).

YAP1 serves as a key regulator of tissue growth and organ size by regulating cell proliferation and apoptosis [38]. To parse out whether EREG could function through activating YAP1, immunofluorescence analysis was performed using the vagina from OVX control and *Rptor* cKO mice (Fig. 6F). As previously reported in mouse epidermis [39], our results suggested that YAP1 is highly expressed in suprabasal-differentiated cell layers in the vaginal epithelium of OVX control mice treated with E2. YAP1 level was significantly decreased in the vaginal epithelium of OVX *Rptor* cKO mice treated with E2. Interestingly, EREG treatment significantly induced the expression level of YAP1 in *Rptor* cKO mice (Fig. 6F). Together, these data showed that EREG could be necessary for full activation of estrogen effects in the vagina. And EREG might promote the differentiation of vaginal epithelium by promoting cell proliferation and maintaining cell survival via YAP1.

### mTORC2 signaling has no effect on the homeostasis of the vaginal epithelium

**Fig. 7:**
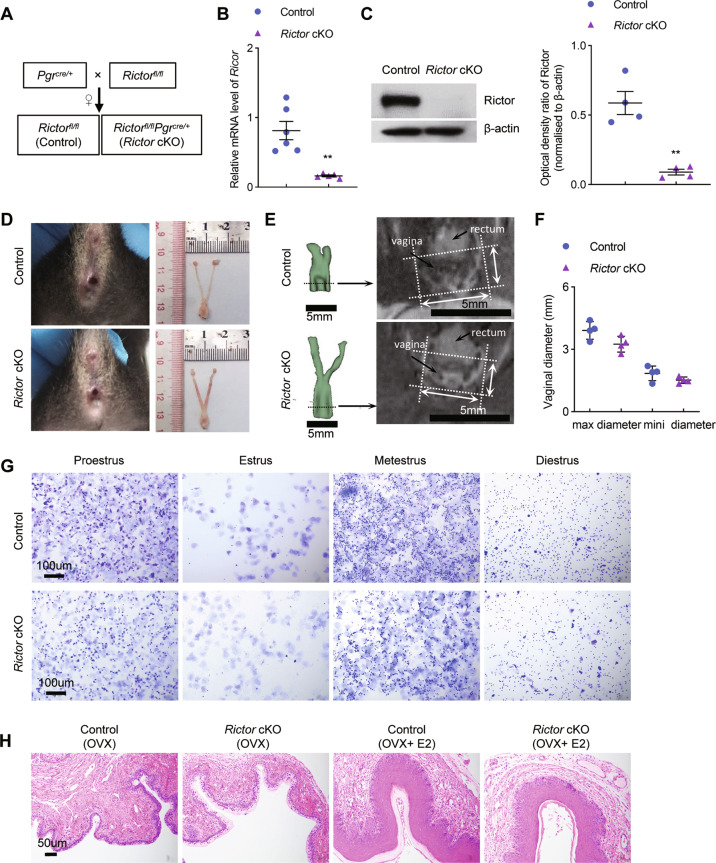
mTORC2 signaling plays no role in the development of vaginal epithelium. **A** The breeding strategy used to generate *Rictor* cKO female mice. **B** Expression of *Rictor* was quantified using qPCR in the vagina of control (*n* = 6) and *Rictor* cKO (*n* = 5) mice. **C** Rictor protein levels in control (*n* = 4) and *Rictor* cKO (*n* = 4) vagina tissues were determined by immunoblotting. β-Actin was used as the loading control. The experiments were repeated two times. **D** Gross morphology of external vaginal orifice and reproductive tracts of control (*n* = 5) and *Rictor* cKO (*n* = 5) mice. **E** 3D renderings of the female reproductive tract in control (*n* = 4) and *Rictor* cKO (*n* = 4) mice based on MRI T2 weighted images. Scale bars: 5 mm. **F** Accurate max and min diameters of the vagina were measured based on **E**. Values are expressed as the mean ± SEM. **G** Representative images for cytological assessment of vaginal smears of control and *Rictor* cKO mice using crystal violet staining method for estrous cycle determination. *n* = 5 mice in each group. Microscopy with magnification ×20. Scale bars: 100 μm. **H** Representative HE-stained vaginal sections of OVX control and *Rictor* cKO mice are shown. Nuclei were stained with hematoxylin. *n* = 3 mice in each group. The experiments were repeated three times. Microscopy with magnification ×20. Scale bars: 50 μm.

It has been reported that mTORC2 contributes to Akt activation, which in turn signals to Foxo1, and acts in synergy with mTORC1 [40]. To explore Rictor/mTORC2-dependent molecular pathway in vaginal epithelial cell homeostasis, mice carrying homozygous alleles of *Rictor* with loxP sites were mated to mice carrying a *Pgr*-Cre allele to generate conditional *Rictor* knockout mice (*Rictor* cKO) (Fig. 7A). The deletion of *Rictor* gene and protein was verified by qPCR and western blot (Fig. 7B, C). Observation of the reproductive duct in *Rictor* cKO mice showed that the external vaginal orifice of *Rictor* deficient mice was comparable with that of control mice (Fig. 7D). MRI technique was also used for the evaluation of vaginal development in vivo. Adult *Rictor* cKO and the control mice had comparable max and min vaginal diameters (Fig. 7E, F). Vaginal smears from *Rictor* cKO mice showed four different stages of the estrous cycle just as control mice (Fig. 7G), indicating *Rictor* cKO mice had normal estrous cycles. HE results showed that the integrity of vaginal tissue in *Rictor* cKO mice was the same as the controls (Fig. 7H). In the OVX model, vaginal epitheliums of *Rictor* cKO mice proliferated and differentiated rapidly following E2 administration, with no difference observed between control and *Rictor* cKO mice (Fig. 7H). These results suggested that mTORC2 signaling does not predominate in E2-mediated proliferation and differentiation of mouse vaginal epithelium.

## Discussion

**Fig. 8:**
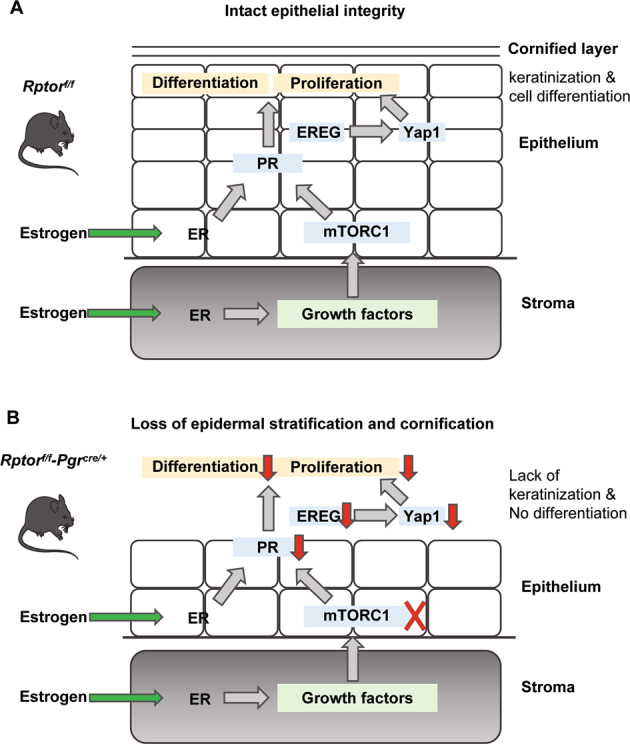
A possible scheme depicting the role of mTORC1 signaling in estrogen-induced epithelial cell proliferation and differentiation in mouse vagina. **A** mTORC1 signaling participates in the proliferation and differentiation of vaginal epithelium by promoting the expression level of PR and EREG-YAP1 in *Rptor*^fl/fl^ mice. **B** Loss of *Rptor* compromises the estrogen-induced proliferation and differentiation of vaginal epitheliums through down-regulating the expression of PR and EREG.

Estrogen plays a regulatory role in the development and functional maintenance of female reproductive organ biology [41]. Although estrogen has been shown to activate mTOR signaling, its role in the development and function of the vagina remains unknown. Using loss-of-function mice models, we characterized the role of mTORC1 signaling in vaginal epithelial homeostasis maintenance. A hypothetical scheme for this study was proposed in Fig. 8. In vaginal tissues, estrogen activates the mTORC1 signaling pathway by secreting growth factors after binding to ER. Activated mTORC1 participates in the proliferation and differentiation of vaginal epithelium by regulating the expression level of PR and EREG-YAP1 (Fig. 8A). Loss of mTORC1 suppresses estrogen reactivity and down-regulates the expression of PR and EREG, thus compromising the estrogen-induced proliferation and differentiation of vaginal epitheliums (Fig. 8B). Our results demonstrated that mTORC1 is necessary for the development and homeostasis of the vaginal epithelium, which might provide new insights into the management of post-menopausal women with vaginal atrophy.

Results from previous studies indicated vaginal estrogen as an effective treatment for symptoms of vaginal atrophy by increasing vaginal tissue thickness and moisture and improving vaginal blood flow [32]. Likewise, two weeks after the ovaries were removed, atrophic changes developed in the vaginal mucosa and vulva. Interestingly, a significant increase of vaginal epithelium thickness after E2 challenge in OVX control mice was observed, but not in *Rptor* cKO mice.

Moreover, estrogen target genes, including *Klk1*, *Pgr*, were significantly upregulated after E2 treatment in the vagina of control mice, but not in *Rptor* cKO mice, implying that the vagina of *Rptor* cKO mice is estrogen insensitive. Of note, our findings are in line with a previous study showing that epithelial PR could contribute to dysregulation of the proliferative response to estrogen in mouse vaginal epithelium [37]. The kallikrein-related peptidase (KLK) family, abundantly expressed in the tissues of the female reproductive tract [42], is tightly regulated by estrogen. KLK1 was proved to promote keratinocyte migration and proliferation via activating protease-activated receptor signaling [43]. Reinforcing estrogen might initiate several signaling cascades including mTOR to promote proliferation and differentiation of the vaginal epithelium.

The coordinated crosstalk between growth factors and hormones is essential for the function and homeostasis of the female reproductive organ. Administration of EGF or TGF-α to OVX adult mice induces cell proliferation and differentiation of female reproductive tracts, while blockade of TGF-α or EGF alone can not completely suppress estrogen-induced mitogenesis [44, 45]. In OVX rats, intravaginal administration of BTC protein results in vaginal epithelial proliferation [32]. Moreover, AREG has been implicated as an effector of ESR1 in mosue vaginal development using an organ culture system [37]. After profiling the mRNA level of growth factors in the vagina of control and *Rptor* cKO mice with or without treatment of E2, we found that *Ereg* and *Btc* were the top two significantly upregulated genes in the vagina of control mice after E2 administration, but not in *Rptor* cKO mice. To investigate whether EREG is involved in the proliferation and differentiation of vaginal epitheliums mediated by mTOR, EREG was administrated locally in the vagina of OVX *Rptor* cKO mice, which could partially rescue the defect of vaginal epithelial differentiation in *Rptor* cKO mice. Interestingly, EREG supplementation failed to promote the expression of PR in the vaginal epithelium (results not shown), although growth factors like EGF and IGF-I act as an inducer of PR [46, 47], suggesting EREG could be a downstream target of PR, consistent with previous reports in mice ovaries [48]. As EREG is a ligand of EGFR and ErbB4, we also profiled the mRNA level of the mouse ErbB family of receptor tyrosine kinases. The qPCR results suggested that the mRNA level of vaginal *Egfr*/*Erbb1*, *Erbb2*, *Erbb3*, and *Erbb4* were comparable between the WT and *Rptor* cKO mice treated with E2 (data not shown). We believe that mTORC1 was involved in the expression of estrogen-induced growth factor EREG, without affecting the levels of erbB receptors in the vagina.

The Hippo/YAP1 signaling pathway is a critical regulator of cell differentiation, proliferation, and apoptosis. Previous studies have proved that YAP1 abundance can be regulated by gonadotropins and sex steroid hormones [49]. E2 exerts pro-growth and anti-apoptotic effects by upregulating the expression of YAP1 in breast cancer cells [50]. Likewise, YAP1 acts an important role in promoting proliferation and inhibiting apoptosis of the endometrium [51, 52]. YAP1 can be activated by ligands of EGF family [53]. Conversely, activation of YAP1 also results in the upregulation of EGF-like growth factors, like AREG [54] and EREG [55]. We found that YAP1 level was significantly elevated after vaginal administration of EREG in *Rptor* cKO mice co-administrated with E2. Therefore, mTORC1 regulates the proliferation and differentiation of estrogen-induced vaginal epitheliums through the EREG-YAP1 pathway.

Of note, the exact role of mTOR in stromal cells and epitheliums can not be accurately clarified by using our mouse models, and more applicable models are needed. For example, epithelial-specific knockout mice models like *CK-5* Cre, as stromal-derived factors might also play an important role in vaginal epithelium homeostasis. Intriguingly, even though a previous study suggested that mTORC1 loss of function does not significantly alter epidermal proliferation or apoptosis in the skin [56], we did observe cell apoptosis and proliferation inhibition in the vaginal epithelium.

In summary, mTORC1 is critical in estrogen-induced proliferation and differentiation of vaginal epitheliums. In the absence of mTORC1, vaginal epitheliums lose estrogen responsiveness, resulting in atrophy. Overall, an improved understanding of downstream molecules involved in estrogen activity at the tissue level will shed light on the mechanisms of estrogen-mediated vagina homeostasis, providing new insights into our understanding of female reproductive disorders.

## Materials and methods

### Mice

*Rptor*^*flox/flox*^ mice (stock number 013191) and *Rictor*^*flox/flox*^ mice (stock number 020649) were purchased from the Jackson laboratory. *PR-Cre* mice were obtained from Cyagen Biosciences (Guangzhou, China). *Rptor*^*flox/flox*^ mice were crossed with *PR-Cre* mice to generate *Rptor*^*fl/fl*^*PR*^*Cre/+*^ (*Rptor* cKO*)* mice*. Rictor*^*flox/flox*^ mice were crossed with *PR-Cre* mice to generate *Rictor*^*fl/fl*^*PR*^*Cre/+*^ (*Rictor* cKO*)* mice*. Rptor*^*flox/flox*^ mice or *Rictor*^*flox/flox*^ mice were used as control. Littermates floxed and gene-deleted mice were utilized within the same set of experiments to minimize the influence of genetic background variability. All mice used in this investigation were housed under the specific pathogen-free animal facilities in the Animal Resource Center at Jinan University, in accordance with the ethical guidelines of the Animal Ethics Committee of Jinan University (Approval No: 20210128-12).

### Crystal violet staining of vaginal smear for mouse estrous cycle staging identification

Phases of the estrous cycle of *Rptor* cKO, *Rictor* cKO, and control mice (8–10 w) were identified using crystal violet staining of vaginal smear as described previously [57]. Briefly, the vaginal cells were flushed by gently introducing 100 μl PBS using a pipette, and transferred to a dry glass slide. The slide was air-dried, stained with 1% crystal violet (V5265-500ML, Sigma) for 1 minute, followed by a 1-minute rinse in water three times. The slides were overlaid with a coverslip using neutral balsam (G8590-100ml, Solarbio), and viewed with Olympus BX53 microscope.

### Measurement of serum estradiol and progesterone levels

Mouse blood samples were collected at the detected time. Serum levels of estradiol-17β (E2) and progesterone (P4) were measured using Estradiol 2 Assay Kit (H102-1, Nanjing Jiancheng Bioengineering Institute, Nanjing, China) and Progesterone Assay Kit (H089, Nanjing Jiancheng Bioengineering Institute, Nanjing, China) according to the manufacturer’s recommendations.

### Hormone treatments

In most experiments, mice were OVX at 6–8 weeks of age and rested for 2 weeks to eliminate the endogenous ovarian hormone. For examining the effects of E2, a single subcutaneous injection of 100 ng E2 (E8875, Sigma) was given to OVX mice for 3 days and killed 24 h after the last injection. Vaginas were collected for histology and qPCR analysis. Vagina from *Rptor* deletion and control mice treated with E2 or PBS were collected for RNAseq analysis. To determine the potential role of E2 in activating mTORC1 signaling, a single subcutaneous injection of 100 ng E2 was given to the OVX mice and sacrificed 6 h after the injection. Vaginas were collected for immunofluorescence of Raptor and Phospho-S6 in Fig. 1D, E. For examining the effect of EREG, an intravaginal injection of 400 ng EREG protein (50599-M01H, Sinobiological) or PBS was given to OVX mice for 3 days, and the mice were sacrificed 24 h after the last injection.

### Histology

Mouse vaginal tissues were fixed in Paraformaldehyde Fix Solution (G1101-500ML, Servicebio) for 24 h and embedded in paraffin, and sectioned at 5 μm. Sections were dried at 60 °C for 30 min, deparaffinized and dehydrated in ddH2O; 75% ethanol, 85% ethanol, 90% ethanol, 100% ethanol, and water. Sections of the vagina were stained with hematoxylin (G1140, Solarbio) and eosin (G1100, Solarbio). Cornification of vaginal epithelial cells was identified using PAS staining (G1281, Solarbio). The TUNEL assay was performed on paraffin-embedded sections of the vagina using an In Situ Cell Death Detection Kit (11684817910, Roche) according to the manufacturer’s instructions.

### Immunofluorescence and immunohistochemistry

Antigen retrieval was carried out by heating the sections in sodium citrate buffer (10 mM sodium citrate, 0.05% Tween 20, pH 6.0) for 15 min. Sections were permeabilized in 0.2% Triton-100 in PBS for 45 min and then blocked with 1% (wt/vol) BSA Fraction V (ST023, Beyotime) and 10% Goat serum (vol/vol) (B900780, Proteintech) in PBS before the primary antibodies were added in immunohistochemical and immunofluorescence staining.

For immunofluorescence staining, sections were incubated with the following primary antibodies: anti-Raptor (sc-81537, Santa Cruz Biotechnology), anti-Phospho-S6 Ribosomal Protein (Ser235/236) (62016, Cell Signaling Technology), anti-PR (9856 S, Cell Signaling Technology), anti-ER-alpha Ab (ab32063, Abcam), anti-Ki67 Ab (ab15580, Abcam), anti-YAP1 Ab (13584-1-AP, Proteintech), anti-EREG Ab (MAB1068, R&D Systems) overnight at 4 °C. Followed by the incubation with the secondary antibodies: Alexa Fluor 488-conjugated affiniPure Goat anti-Rabbit IgG (H + L) (115-585-146, Jackson ImmunoResearch), Alexa Fluor 594-conjugated affiniPure Goat anti-Mouse IgG (H + L) (111-545-144, Jackson ImmunoResearch) for 1 h, and nuclei-staining with DAPI (D9542, Sigma) for 10 min at room temperature. Pictures were taken using Leica TCS SP8 confocal microscope.

For immunohistochemistry, the tissue sections were immersed in 3% H_2_O_2_ for 45 min at room temperature to quench endogenous peroxidase after permeabilization. And the following primary antibodies were used: anti-Ki67 Ab (ab15580, Abcam) overnight at 4 °C. On the following day, the sections were incubated with biotinylated secondary antibodies (BA-1000-1.5, Vector Laboratories) for 1 hour at room temperature. After several rinses in PBS, the sections were incubated with the Vectastain Elite ABC reagent (PK-6100, Vector Laboratories) for 30 min, and immunoreactive signals were developed using ImmPACT DAB EqV Peroxidase (HRP) Substrate (SK-4103, Vector Laboratories), and counterstained with hematoxylin.

### RNA extraction and qPCR

Total RNA was extracted from vagina tissues using TRIzol reagent (Invitrogen, Carlsbad, CA, USA) according to the manufacturer’s protocol. The complementary DNAs (cDNAs) were synthesized with PrimeScript RT Master Mix (RR036A, Takara) by using 500 ng total RNAs according to the manufacturer’s instructions. Quantitative real-time PCR was performed to assess the expression of genes of interest with SYBR Green (RR820A, Takara) on a CFX Connect Real-Time PCR Detection System. The expression levels of mRNA were determined by the comparative cycle threshold (CT) (2−∆∆Ct) methods. Experimental gene expression data were normalized by *Actb*. The qPCR primers are listed in Supplemental Table 1.

### Western blot

Protein extraction and western blot analysis were performed as previously described [58]. Antibodies against Raptor (sc-81537, Santa Cruz Biotechnology), Rictor (2140 S, Cell Signaling Technology), PR (9856 S, Cell Signaling Technology), ER-alpha (ab32063, Abcam), and β-actin (66009-1-Ig, Proteintech) were used.

### Superovulation assay

Female mice (8 weeks) were administrated 7.5 IU of pregnant mare serum gonadotropin (PMSG, hor-272, ProSpec), followed 48 h later by 7.5 IU of human chorionic gonadotropin (hCG, 230734, sigma) (i.p.). After 14 h of hCG injection, the ovaries and oviducts were surgically removed and the cumulus-oocyte complexes mass was recovered from the oviduct and collected into M2 medium (Sigma) containing 1 mg/mL of hyaluronidase (H3506, Sigma) to dissociate the cumulus cells from oocytes. The numbers of oocytes were counted and recorded.

### In vivo MRI image acquisition

The MRI study was performed using a Bruker Biospec 9.4 T preclinical MRI scanner (Bruker Corp., USA) equipped with a 35 mm inner diameter mouse body radiofrequency coil. T2 weighted images of the murine female reproductive tract, including vagina and uterus, were acquired using the parameters summarized in Supplemental Table 2. Visualization, segmentation, and the 3D model establishment of murine female reproductive tract were performed using 3D Slicer (version 4.11.20210226). Cross-sectional diameters of the vagina were measured using the ruler function of 3D Slicer.

### Gene set enrichment analysis

Previously published human data (accession number: GSE11622 and GSE26761) were used for microarray analysis and GSEA analysis. The data were normalized using limma R package. GSEA analysis was performed using clusterProfiler R package.

### RNA sequencing analysis

Total RNA was extracted from vagina tissues using TRIzol reagent (Invitrogen) following the manufacturer’s protocol. RNA purity and quantification were evaluated using the NanoDrop 2000 spectrophotometer (Thermo Scientific, USA). RNA integrity was assessed using the Agilent 2100 Bioanalyzer (Agilent Technologies, USA). Libraries were constructed using TruSeq Stranded mRNA LT Sample Prep Kit (Illumina, USA) and sequenced on a Novaseq6000 platform. The clean reads were mapped to the mouse reference genome (GRCm38.p6) using HISAT2. FPKM of each gene was calculated using Cufflinks, and the read counts of each gene were obtained by HTSeq-count. Differential expression analysis was performed using the DESeq (2012) R package. *q* value <0.05 and foldchange >2 or foldchange <0.5 was set as the threshold for significantly differential expression. The transcriptome sequencing and analysis were conducted by OE Biotech Co., Ltd. (Shanghai, China). The RNAseq data were deposited in the Sequence Read Archive (SRA) repository at NCBI under the BioProject ID PRJNA787772.

### Statistics

Statistical analyses were performed using two-tailed Student’s *t* test. *P* values < 0.05 were considered statistically significant. *p < 0.05, **p < 0.01, and ***p < 0.001.

## Supplementary information

SUPPLEMENTAL DATA

Original Data File

Checklist

## Data Availability

All data needed to evaluate the conclusions in the paper are present in the paper and/or the Supplementary Materials. The RNAseq data were deposited in the SRA repository at NCBI under the BioProject ID PRJNA787772. Additional data related to this paper may be requested from the authors.
