# Supplementary material for: mTORC1 signaling pathway integrates estrogen and growth factor to coordinate vaginal epithelial cells proliferation and differentiation
Source: Cell Death Dis. 2022 Oct 11;13(10):862. doi: 10.1038/s41419-022-05293-8 (PMC9553898; doi:10.1038/s41419-022-05293-8)
Supplement: Supplementary file 1 — SUPPLEMENTAL DATA [file 41419_2022_5293_MOESM1_ESM.docx]

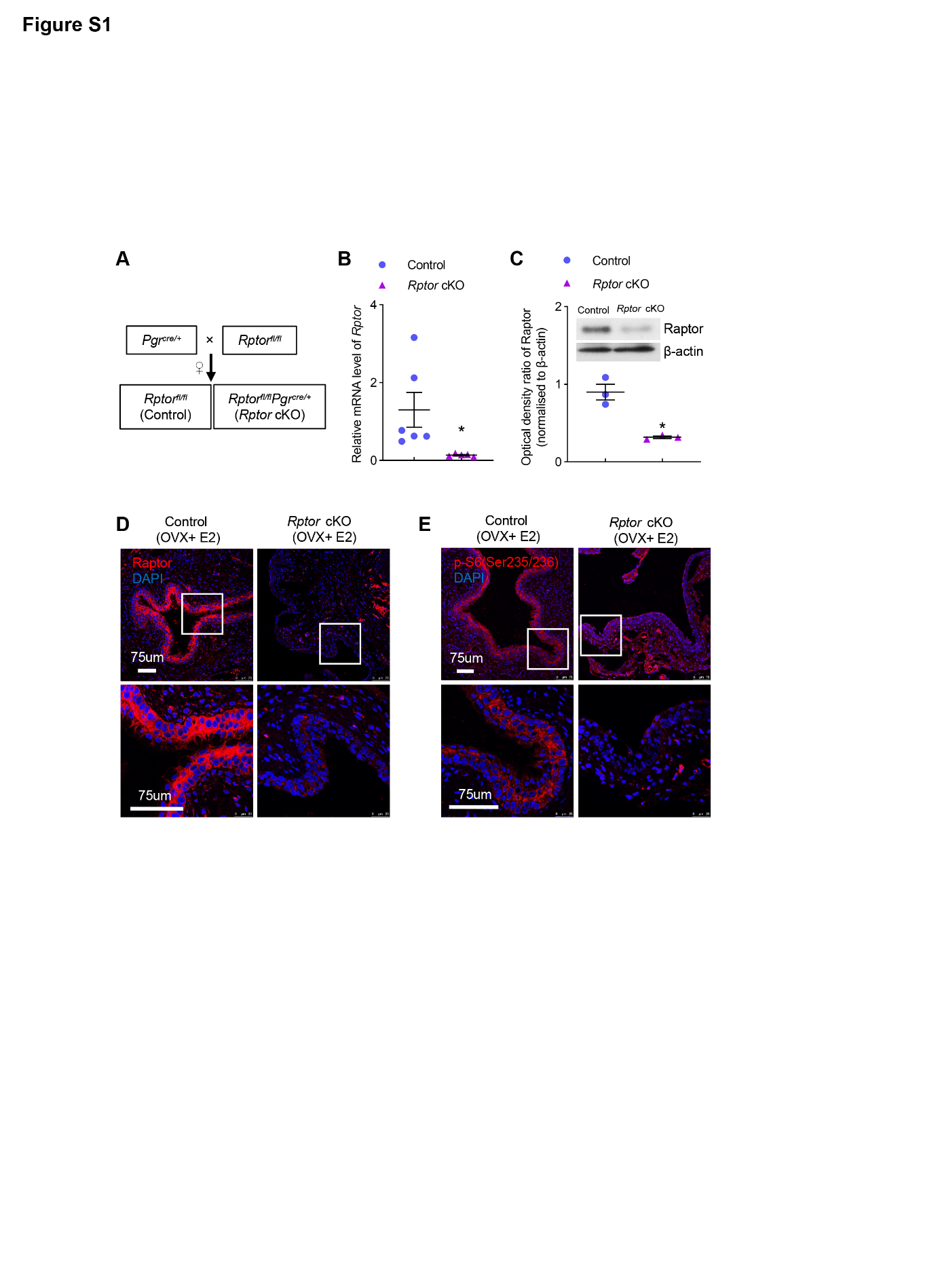


**Supplemental Figure 1. Generating mice with conditional deletion of *Rptor*.** (A). The breeding scheme used to generate *Rptor* cKO female mice. **(B)**. Expression of *Rptor* was quantified using qRT-PCR in the vagina of control (n=6) and *Rptor* cKO (n=5) mice. Values are expressed as the mean ± SEM. **p* < 0.05. **(C)**. Immunoblotting analysis of Raptor protein levels in control (n=3) and *Rptor* cKO (n=3) vagina. The experiments were repeated 3 times. β-actin was used as the loading control. Values are expressed as the mean ± SEM. **P* < 0.05. **(D and E)** Representative images of the Raptor (**D**) or p-S6(Ser235/236) (**E**) immunofluorescence staining in the vagina of OVX control (n=3) and *Rptor* cKO (n=3) mice 6 hours after E2 administration. The experiments were repeated 3 times. Nuclei were stained with DAPI. Microscopy with magnification ×20 (Upper) and ×63 (Lower). Scale bars: 75 μm. **(B and C)**

Supplemental Table 1. List of primer sequences for qPCR.

| Primer name | Sequences(5'-3') |
| --- | --- |
| *Rptor* Forward | TTCACATCCTGCCTCACCAC |
| *Rptor* Reversed | CCGGCCAGGGATCTTTTCTA |
| *Krt6a* Forward | TCTCAACATCATAACCCTCCCTG |
| *Krt6a* Reversed | ATTACGAGGAAGCCAAGAGCA |
| *Krt6b* Forward | GGCCTGGGAAACCCCATT |
| *Krt6b* Reversed | AAGGAAACAAAGAGCAGAGATGG |
| *Krt10* Forward | GGAAGCCTCCTTGGCAGAAA |
| *Krt10* Reversed | TCTGTTGCAGCTGTTCCTCC |
| *Krt13* Forward | CAAAACAAGGCTGGAACAGGAG |
| *Krt13* Reversed | CCCGTTGGAGGTAGTAGTGTT |
| *Krt16* Forward | TATCCACAGCTCCTCCTCACA |
| *Krt16* Reversed | TGGTTGAACCTTGCTCCTTGA |
| *Pgr* Forward | TATGAGAACCCTTGACGGTGTTG |
| *Pgr* Reversed | CAGGGCCTGGCTCTCGTT |
| *Klk1* Forward | GGTATCCTGCTGAACGCCAACT |
| *Klk1* Reversed | TGTTGGGCAGAGGGTTCATCCT |
| *Areg* Forward | ACAGCGAGGATGACAAGGAC |
| *Areg* Reversed | GCCAATAGCTGCGAGGATGA |
| *Gab1* Forward | ACGGACCTTTCCGAGCGATAGA |
| *Gab1* Reversed | GGAACGTAGTTCTCATCCAGCTC |
| *Ereg* Forward | TGCTTTGTCTAGGTTCCCACC |
| *Ereg* Reversed | GGCGGTACAGTTATCCTCGG |
| *Mapk3* Forward | GGCTTTCTGACGGAGTATGTGG |
| *Mapk3* Reversed | GTTGGAGAGCATCTCAGCCAGA |
| *Rps6kb2* Forward | TGACAGAGAGCAGCGTGAACCT |
| *Rps6kb2* Reversed | GCCTTGCACTTTTCTCACCTGG |
| *Btc* Forward | TTCGTGGTGGACGAGCAAACTC |
| *Btc* Reversed | CCATGACCACTATCAAGCAGACC |
| *Mapk2k1* Forward | AAGGTCTCCCACAAGCCATCTG |
| *Mapk2k1* Reversed | AGTTGCACTCGTGCAGTACCTG |
| *Mapk2k4* Forward | GTGATTTCGGCATCAGTGGACAG |
| *Mapk2k4* Reversed | CCAAACTCCAGACATCAGAGCG |
| *Mycbp* Forward | TGCTGGACACGCTGACGAAAGT |
| *Mycbp* Reversed | GCCAATTCTAGGCGAAGCAGCT |
| *Hras* Forward | TCGCACTGTTGAGTCTCGGCAG |
| *Hras* Reversed | TATGCTGCCGAATCTCACGGAC |
| *Pak6* Forward | TCCGAAGCATGTTCCTGTCCAC |
| *Pak6* Reversed | GCTACCAGGTTTGAAGAGGAGTC |
| *Mapk2k2* Forward | ATCTGCATGGAGCACATGGAT |
| *Mapk2k2* Reversed | CCCGGAGCACCGCAA |
| *Erbb1* Forward | GCCATCTGGGCCAAAGATACC |
| *Erbb1* Reversed | GTCTTCGCATGAATAGGCCAAT |
| *Erbb2* Forward | TGAGGGTCGCTACACCTTTG |
| *Erbb2* Reversed | TGACCTCTTGGTTGTTCGGG |
| *Erbb3* Forward | TCTGTGTTGCCAGTTGTCCC |
| *Erbb3* Reversed | TCTGCAAGGCTCACACATCTT |
| *Erbb4* Forward | CAAAGCCAACGTGGAGTTCATGG |
| *Erbb4* Reversed | CTGCGTAACCAACTGGATAGTGG |
| *Rictor* Forward | CTGACGCCAAGCAGGTTTATG |
| *Rictor* Reversed | GGAGCGCTGGAGGGTATTG |
| *Actb* Forward | CACTGTCGAGTCGCGTCC |
| *Actb* Reversed | CGCAGCGATATCGTCATCCA |

Supplemental Table 2. Sequence Parameters for T2-Weighted Turbo-Spin-Echo Sequence

| **Sequence** | **Parameters** |
| --- | --- |
| Repetition Time | 3650 ms |
| Echo Time | 22 ms |
| Echo Train Length | 8 |
| Flip Angle | 180° |
| Number of Averages | 4 |
| Slice Thickness | 0.5 mm |
