# Supplementary figures and images for: mTORC1 signaling pathway integrates estrogen and growth factor to coordinate vaginal epithelial cells proliferation and differentiation
Source: Cell Death Dis. 2022 Oct 11;13(10):862. doi: 10.1038/s41419-022-05293-8 (PMC9553898; doi:10.1038/s41419-022-05293-8)

Figure 5A

PR-B  
PR-A

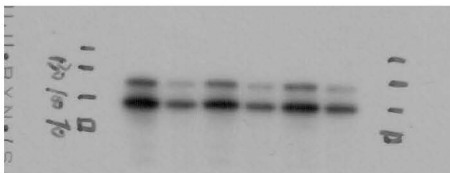

ER

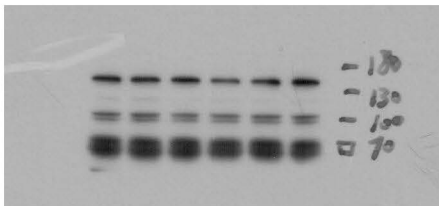

beta-actin

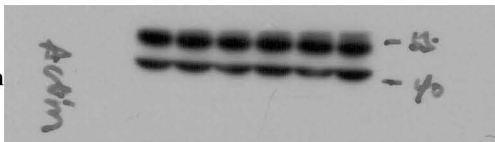

Figure 7C

**Rictor**

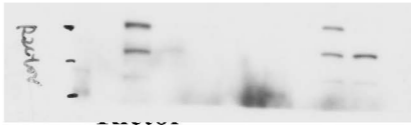

**beta-actin**

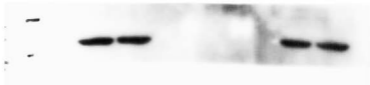

**Raptor**

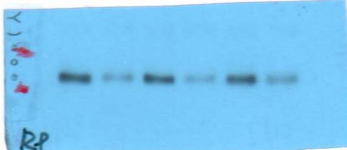

**beta-actin**

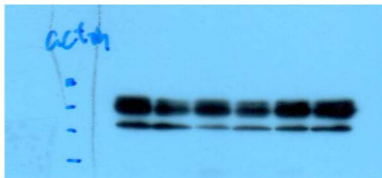

Supplement: Supplementary file 2 — Original Data File [file 41419_2022_5293_MOESM2_ESM.pdf]
